# Substrate Reduction Therapy Reverses Mitochondrial, mTOR, and Autophagy Alterations in a Cell Model of Gaucher Disease

**DOI:** 10.3390/cells10092286

**Published:** 2021-09-02

**Authors:** Yanyan Peng, Benjamin Liou, Yi Lin, Venette Fannin, Wujuan Zhang, Ricardo A. Feldman, Kenneth D. R. Setchell, Gregory A. Grabowski, Ying Sun

**Affiliations:** 1 Division of Human Genetics, Cincinnati Children’s Hospital Medical Center, Cincinnati, OH 45229, USA; Yanyan.Peng@cchmc.org (Y.P.); Benjamin.Liou@cchmc.org (B.L.); Yi.Lin@cchmc.org (Y.L.); Venette.Davis@cchmc.org (V.F.); grabgo317@comcast.net (G.A.G.); 2 Department of Pathology, Clinical Mass Spectrometry Laboratory, Cincinnati Children’s Hospital Medical Center, Cincinnati, OH 45229, USA; Wujuan.Zhang@cchmc.org (W.Z.); kenneth.setchell@cchmc.org (K.D.R.S.); 3 Department of Microbiology and Immunology, University of Maryland School of Medicine, Baltimore, MD 21201, USA; RFeldman@som.umaryland.edu; 4 Department of Pediatrics, University of Cincinnati College of Medicine, Cincinnati, OH 45229, USA

**Keywords:** lysosomal storage disorders, cell biology, neurons, glucosylceramide, glucosylsphingosine

## Abstract

Substrate reduction therapy (SRT) in clinic adequately manages the visceral manifestations in Gaucher disease (GD) but has no direct effect on brain disease. To understand the molecular basis of SRT in GD treatment, we evaluated the efficacy and underlying mechanism of SRT in an immortalized neuronal cell line derived from a *Gba* knockout (*Gba*^-/-^) mouse model. *Gba*^-/-^ neurons accumulated substrates, glucosylceramide, and glucosylsphingosine. Reduced cell proliferation was associated with altered lysosomes and autophagy, decreased mitochondrial function, and activation of the mTORC1 pathway. Treatment of the *Gba*^-/-^ neurons with venglustat analogue GZ452, a central nervous system-accessible SRT, normalized glucosylceramide levels in these neurons and their isolated mitochondria. Enlarged lysosomes were reduced in the treated *Gba*^-/-^ neurons, accompanied by decreased autophagic vacuoles. GZ452 treatment improved mitochondrial membrane potential and oxygen consumption rate. Furthermore, GZ452 diminished hyperactivity of selected proteins in the mTORC1 pathway and improved cell proliferation of *Gba*^-/-^ neurons. These findings reinforce the detrimental effects of substrate accumulation on mitochondria, autophagy, and mTOR in neurons. A novel rescuing mechanism of SRT was revealed on the function of mitochondrial and autophagy–lysosomal pathways in GD. These results point to mitochondria and the mTORC1 complex as potential therapeutic targets for treatment of GD.

## 1. Introduction

Gaucher disease (GD) is caused by mutations in *GBA1*-encoding acid β-glucosidase (GCase, EC3.2.1.45), the lysosomal enzyme responsible for degradation of the glycosphingolipids, glucosylceramide (GC), and glucosylsphingosine (GS), a deacylated form of GC [1]. The prevalence of GD in general population is ~1/60,000, and in the Ashkenazi Jewish population it is 1 in 450. GD phenotypes are classified as non-neuropathic (Type 1, GD1) or neuronopathic (Type 2, GD2 and Type 3, GD3) diseases depending on the clinical manifestations [2,3]. About 10% of patients in the US and Europe and 75% in Asian countries present with GD2 or GD3 [2,4,5,6]. Typical manifestations of GD1 include hepatosplenomegaly, anemia, and thrombocytopenia as well as boney abnormalities, which are effectively treated with enzyme replacement therapy (ERT) or substrate reduction therapy (SRT). GD2 is an acute, rapidly progressive currently untreatable neonatal or early post-natal central nervous system (CNS) degenerative disease leading to death by 2 years of age [7]. GD3 is a subacute CNS and visceral disease presenting a highly variable phenotype ranging from progressive CNS disease early in childhood to adulthood, with death caused by currently untreatable CNS disease [8]. Neuronopathic GD (nGD) affects the CNS through progressive neurodegeneration and neuroinflammation, leading to functional deficits and mortality [2,8,9,10]. Mutations in *GBA1* have been identified as the most common genetic risk factor for developing earlier onset and more rapidly progressive Parkinson’s disease, even in those who have only one mutant *GBA1* allele [11]. In GD, cellular functions are disrupted by *GBA1* mutations and substrate accumulations. Abnormal autophagy, either increased or decreased, has been demonstrated in nGD cell and animal models and in patient brain samples [12,13]. Furthermore, mitochondrial dysfunction is a well-documented pathological feature in chemically induced cell and genetic mouse models of nGD [14,15,16,17]. Recently, mTOR pathway hyperactivity has also been implicated in nGD iPSC-derived neurons [18,19]. Accumulations of GC and GS have been considered a primary insult leading to GD manifestations, particularly in nGD, and may directly alter cellular functions [3].

SRT via inhibition of glucosylceramide synthase is an approved approach for treating GD. Two SRT drugs (eliglustat and miglustat) are being used to treat GD1 [20]. Because eliglustat does not access the CNS and miglustat has questionable efficacy in the brain, the CNS-accessible small molecule drug venglustat was developed. A Phase III clinical trial of venglustat showed promise in improving biochemical and phenotypic parameters in GD3 patients [21]. An analogue of venglustat, GZ-682452 (termed herein as GZ452), was effective at attenuating several neuropathologic and behavioral manifestations associated with nGD mouse models [22,23,24]. However, the underlying mechanism(s) of SRT in protecting cellular functions are poorly understood. In the present study, cellular function in a neuronal model of GD (*Gba*^-/-^ neurons) completely lacking GCase identified compromised autophagic activity, impaired mitochondrial function, and hyperactivity of the mTOR pathway associated with GC and GS accumulations. The effect of SRT with GZ452 on these cellular functions was evaluated in these neurons.

## 2. Materials and Methods

### 2.1. Cell Culture and Brain Tissues

The immortalized neurons were provided by Dr. Ellen Sidransky [25]. *Gba*^-/-^ and *Gba*^+/+^ neurons were derived from 17-day-old embryonic null allele *Gba*^-/-^ mice and control littermate *Gba*^+/+^ mice, respectively [25]. Cultured primary cortical neurons from mouse embryo brains were immortalized by infection with an EF1α-SV40T lentivirus and isolated by fluorescence-activated cell sorting using neuronal marker CD24. The immortalized *Gba*^+/+^ and *Gba*^-/-^ neurons were grown in neurobasal growth medium (500 mL neurobasal medium, 1:50 (*v*/*v*) B27, 1:200 (*v*/*v*) glutamine, and 25 mM HEPES (Life Technologies)). The cells were cultured in 3 mL medium including 500 µL of cell suspension and 2.5 mL of neurobasal growth medium per well on a 6-well plate pre-coated with poly-L-lysine (Sigma Aldrich, St. Louis, MO, USA). The medium was changed every 3 days by replacing 50% medium with fresh neurobasal growth medium [25]. For SRT treatment, the *Gba*^-/-^ neurons were treated for 5 days with GZ452 (P14969, Astatech Inc., Bristol, PA, USA,) at 0, 75, 150, and 300 nM for the dosing determination experiment or 300 nM for biological assays. 

*Gba^-/-(NestinCre)^* and wild-type (WT) mice in C57BL/6 strain background were housed under pathogen-free conditions in the animal facility according to IACUC-approved protocol (2018-0056) at Cincinnati Children’s Hospital Medical Center (CCHMC). The *Gba^-/-(NestinCre)^* line was provided by Dr. Stefan Karlsson and Dr. Tony Futerman [26]. Mouse brain tissues were collected after transcardial perfusion with saline and kept frozen for further analysis. Postmortem frozen human brain tissues were provided by the Discover Together Biobank at Cincinnati Children’s Research Foundation. 

### 2.2. Western Blot (WB) Analyses

The neurons and tissues were lysed by protease and phosphatase cocktail containing RIPA buffer and treated with loading buffer for electrophoresis. The proteins were resolved on 4–12% NuPAGE or 3–8% NuPAGE gel (Invitrogen, Waltham, MA, USA) and then transferred to PVDF membrane using an iBlot 2 gel transfer device (Life Technologies, Carlsbad, CA. USA) following the manufacturer’s instructions [27]. The blotted membranes were incubated with primary antibodies as below: rabbit anti-Phospho-mTOR (1/1000, 5536S, Cell signaling Technology (CST), Danvers, MA, USA), rabbit anti-mTOR (1/1000, 2983S, CST), rabbit anti-Phospho-S6-RP (1/1000, 4856S, CST), rabbit anti-S6 ribosomal protein (S6-RP) (1/1000, 2217S, CST), rabbit anti-Phospho-4E-BP1 (1/1000, 2855S, CST), rabbit anti-4E-BP1 (1/1000, 9452S, CST), rabbit anti-VDAC (1/1000, 4661S, CST), rat anti-Lamp1 (1/1000, OR-CD107abMS, Fitzgerald Industries International, Acton, MA, USA), mouse anti-β-actin (1/5000, MAS-15739, Invitrogen), rabbit anti-LC3B (1/1000, NB100-2220, Novus Biologicals, Littleton, CO, USA), mouse anti-Tom20 (1/1000, MilliporeSigma, Burlington, MA, USA MABT166), rabbit anti-Tom40 (1:1000, MABT166, MilliporeSigma), rabbit anti-TFEB (1/1000, MBS855552, MyBiosource, San Diego, CA, USA), and rabbit anti-Phospho-TFEB (Ser142) (1/1000, ABE1971, MilliporeSigma) in 1.5% BSA/1.5% milk/PBS buffer overnight at 4 °C. After multiple washes in PBS-T (0.5%), PVDF membranes were then incubated with IR dye-labelled secondary antibodies (1/5000 ~ 1/10,000, LI-COR Biosciences, Lincoln, NE, USA) at RT for 1 h. The signals were detected using a LI-COR detection system according to manufacturer’s instructions. 

### 2.3. GCase Activity Assay

The neurons were lysed in 1% sodium taurocholate/1% Triton X-100 (Tc/Tx). GCase activity was determined fluorometrically with the 4MU-Glc substrate in 0.25% Tc/Tx diluted in 0.1M citrate phosphate (CP) buffer (pH 5.6) [28]. Protein concentrations were determined by BCA assay using BSA as standard.

### 2.4. Glycosphingolipid Analyses

Frozen neuron cells were homogenized in water/chloroform/methanol, and glycosphingolipids were extracted as described previously [29]. Aliquots of cell lipids extracts were processed for GC and GS quantification by LC/MS-MS at Clinical Mass Spectrometry Laboratory in CCHMC. The GC and GS levels were normalized to mg of cell protein, determined by BCA assay [29].

### 2.5. Oxygen Consumption Rate Assay

The neurons were plated in XF 96-well plates, and mitochondrial oxygen consumption rate (OCR) was measured as described previously [30]. The data were analyzed using the XFe Wave software. Basal respiration, ATP production, maximal respiration, proton leak, and non-mitochondrial respiration rates in neurons were normalized to cell number.

### 2.6. LysoTracker and MitoTracker Staining

*Gba^+/+^* and *Gba*^-/-^ neurons were plated in coverslips coated with Poly-Lysine and grown to 80% confluency. The cells were given fresh neurobasal growth medium 24 h before adding LysoTracker or MitoTracker. On the day of analysis, cells were incubated with neurobasal medium supplemented with 1 μM LysoTracker Red DND-99 (L7528, Thermo Fisher Scientific, Waltham, MA, USA) or 250 nM MitoTracker Red (M7512, Invitrogen) for 1 h at 37 °C and washed with 1× PBS. Cells were fixed with 4% paraformaldehyde for 5 min followed by 3 times washes with PBS. Cells were counterstained with 300 nM DAPI (Life Technologies, Carlsbad, CA, USA). Images were taken using a Nikon C2+ confocal microscope with Nikon NIS-elements software (version 4.6). Exposure used identical settings for all images. Fluorescence images were exported in TIFF format for quantification analysis using ImageJ2 software (Fiji win 64, v1.51). Quantification of fluorescence signals was calculated based on acquired fluorescent intensity of LysoTracker (Excitation ~577, Emission ~590 nm) or MitoTracker (Excitation ~579, Emission ~599 nm) and normalized with the fluorescent intensity of DAPI (Excitation ~405, Emission ~420 nm) in the same image. The data are from >5 images per sample.

### 2.7. CYTO-ID^®^ Autophagy Dye Staining

Autophagy was measured using CYTO-ID^®^ autophagy detection kit (ENZ-KIT175-0050, Enzo Life Sciences, Inc. Farmingdale, NY, USA). The neurons were grown on Poly-Lysine-coated 96-well black plates with clear bottoms. When the cells reached 90% confluence, the medium was removed and replaced with 100 µL per well of Microscopy Dual Detection Reagent containing 1 µL of CYTO-ID^®^ Green Detection Reagent 2 and 1 µL Hoechst 33342 Nuclear Stain mixed in 1 mL neurobasal medium without Phenol Red Indicator, supplemented with 5% FBS. The cells were protected from light and incubated for 30 min at 37 °C and then washed with 100 µL neurobasal medium supplemented with 5% FBS and without Phenol Red Indicator. The plate was read on a fluorescence microplate reader (SpectraMax M5, Molecular Devices, San Jose, CA, USA). The CYTO-ID^®^ Green detection reagent was read with a FITC filter set (excitation ~480 nm, emission ~530 nm), and the Hoechst 33342 Nuclear Stain was read with a DAPI filter set (excitation ~340 nm, emission ~480 nm). 

### 2.8. Cell Proliferation and Viability Assessment

Cell proliferation and viability were assessed with MTT (3-(e,5-dimethythiazol-2-yl)-2,5-diphenyltetrazolium bromide) assays (Vybrant@ MTT cell proliferation assay kit, V-13154, Molecular Probes, Eugene, OR, USA). The neurons at the logarithmic growth phase were collected and seeded in 96-well tissue culture plates (5 × 10^3^ cells/well) and allowed to adhere overnight. After the cells were cultured in neurobasal growth medium for 1, 2, 3, 4, 5, or 6 days, 20 μL MTT (5 mg/mL) was added into each well. The cells were incubated at 37 °C for 4 h, followed by adding 150 μL of dimethyl sulfoxide and incubated for additional 1 h at 37 °C. The plate was read at absorbance 570 nm using a Synergy HT plate reader (Bio-Tek Instruments, Winooski, VT, USA). 

### 2.9. Mitochondrial Membrane Potential Assay

Neuron cells were plated in a 96-well plate and cultured overnight. The medium was replaced with fresh neurobasal medium with 200 nM tetramethylrhodamine, ethyl ester (TMRE, 13296S, CST) added to each well and incubated at 37 °C for 20 min. For the control group, 50 µM carbonyl cyanide m-chlorophenyl hydrazone (CCCP) was added to the cells and incubated at 37 °C for 15 min. Before reading, the solution was removed, and the plate was washed three times by 1× PBS. Samples were analyzed at excitation of 550 nM and emission of 580 nM on a fluorescence microplate reader (SpectraMax M5, Molecular Devices, San Jose, CA, USA). 

### 2.10. Mitochondrial Isolation

Freshly harvested neurons (about 1 × 10^8^ cells) were washed with PBS and resuspended in 1 mL ice-cold Lysis Buffer (Cat#130-096-946, Miltenyi Biotech, Auburn, CA, USA). After homogenization of the cells with a Dounce homogenizer, cell lysates were transferred to 15 mL conical tubes and mixed with 9 mL of ice-cold 1 × Separation Buffer with 50 µL Anti-TOM22 MicroBeads to magnetically label the mitochondria. The mixture was incubated for 1 h at 4 °C with gentle shaking. An LS Column for separation was placed in the magnetic field of a MACS Separator (MidiMACS Separation Unit) and rinsed with 3 mL of 1× Separation Buffer. The labeled cell lysates were applied onto the column stepwise (3 × 3.3 mL) and the lysate was allowed to run through. The column with the lysate was removed from the MACS Separator and placed on a 15 mL conical tube. An amount of 1× Separation Buffer (1.5 mL) was loaded on the column and immediately flushed out the magnetically labeled mitochondria by firmly pushing the plunger into the column. Isolated mitochondria were collected by centrifuging at 1000 rpm for 1 min.

### 2.11. Electron Microscope (EM)

Neurons were postfixed in EM fixative buffer, washed in 0.1 M sodium cacodylate buffer (EMS; Hatfield, PA, USA), and postfixed in 1% osmium tetroxide (EMS) for 1  h at 4  °C [31]. The fixed cells were washed in 0.1 M sodium cacodylate buffer and dehydrated through a graded ethanol series and embedded in LX-112 (Ladd Research Industries; Williston, VT, USA). The sample blocks were sectioned (thickness, 0.5–1 μm) and cut into 90 nm thick sections with an ultramicrotome (Leica EM UC7; Buffalo Grove, IL, USA). The ultra-thin sections were counterstained with uranyl acetate 2% (EMS) and lead citrate. All images were taken with an 80 kV transmission electron microscope (Hitachi, H-7650, V01.07; Tokyo, Japan). 

### 2.12. Statistical Analyses

Results are presented as means  ±  SEM. The data were analyzed by Student’s *t*-test or one-way ANOVA followed by Tukey post hoc tests using GraphPad Prism 8. A *p*  <  0.05 was considered statistically significant.

## 3. Results

### 3.1. SRT Corrected Substrate Accumulation in Gba^-/-^ Neurons

**Figure 1:**
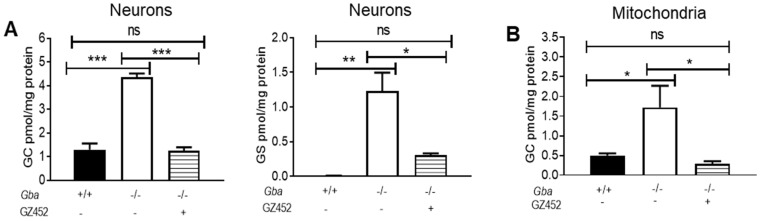
Glucosylceramide (GC) and glucosylsphingosine (GS) analysis by LC/MS-MS in neurons and isolated mitochondria. (**A**) GC and GS levels in *Gba*^-/-^ neurons were significantly reduced by 300 nM GZ452 treatment. (**B**) In the isolated mitochondria, GC level in GZ452-treated *Gba*^-/-^ neurons was reduced to WT (*Gba^+/+^)* level. Triplicate experiments (*n* ≥ 2–4 samples). One-way ANOVA (* *p* < 0.05; ** *p* < 0.01; *** *p* < 0.001). ns, not statistically significant.

*Gba*^-/-^ and *Gba*^+/+^ neurons were derived from 17-day-old embryonic null allele *Gba*^-/-^ mice and *Gba*^+/+^ mice, respectively [25]. The *Gba*^-/-^ neurons lacked GCase activity (Figure S1) and exhibited increased GC and GS, which was associated with enlarged lysosomes [25]. To determine the effect of SRT in reducing GC and GS and lessening the disease phenotypes, *Gba*^-/-^ neurons were treated with GZ452. Optimal concentrations of GZ452 were determined in a dose–response study (Figure S2). A dose of 300 nM that reduced these substrates to levels close to levels in *Gba*^+/+^ neurons but did not affect cell growth during 5 days of treatment was selected for further experiments. In comparison with *Gba*^+/+^, GC levels in *Gba*^-/-^ neurons were 4.5-fold (4.35 pmol/mg) increased, and 300 nM GZ452 treatment potently reduced GC levels in these neurons (1.25 pmol/mg) to levels comparable with *Gba*^+/+^ neurons (0.97 pmol/mg) (Figure 1A). GS accumulated in *Gba*^-/-^ (1.23 pmol/mg) compared with negligible levels in *Gba*^+/+^ (0.01 pmol/mg) neurons. GZ452 treatment reduced GS levels by 75% to 0.315 pmol/mg in *Gba*^-/-^ neurons (Figure 1A). Thus, GZ452 treatment reduced the levels of both GC and GS in *Gba*^-/-^ neurons. 

GD brains exhibit mitochondrial dysfunction [15,16,17]. To determine if mitochondria had excess substrates, these organelles were isolated from *Gba*^-/-^ and *Gba*^+/+^ neurons using anti-Tom20 antibody-conjugated magnetic beads. The purity of isolated mitochondria was confirmed by positivity for the mitochondrial proteins VDAC, Tom20, and Tom40 and negativity for the lysosomal protein Lamp1 (Figure S3). In *Gba*^-/-^ mitochondria, GC levels (3.44 pmol/mg) were about 7-fold higher than those in *Gba*^+/+^ mitochondria (0.50 pmol/mg). GZ452 treatment reduced GC in *Gba*^-/-^ mitochondria (0.29 pmol/mg) to the levels found in *Gba^+/+^* mitochondria (Figure 1B). GS in the isolated mitochondria was below the level of detection. These results confirmed that GZ452 treatment normalized the GC level in *Gba*^-/-^ neurons and isolated neuronal mitochondria.

### 3.2. SRT Improved Oxygen Consumption Rate and Mitochondrial Membrane Potential

**Figure 2:**
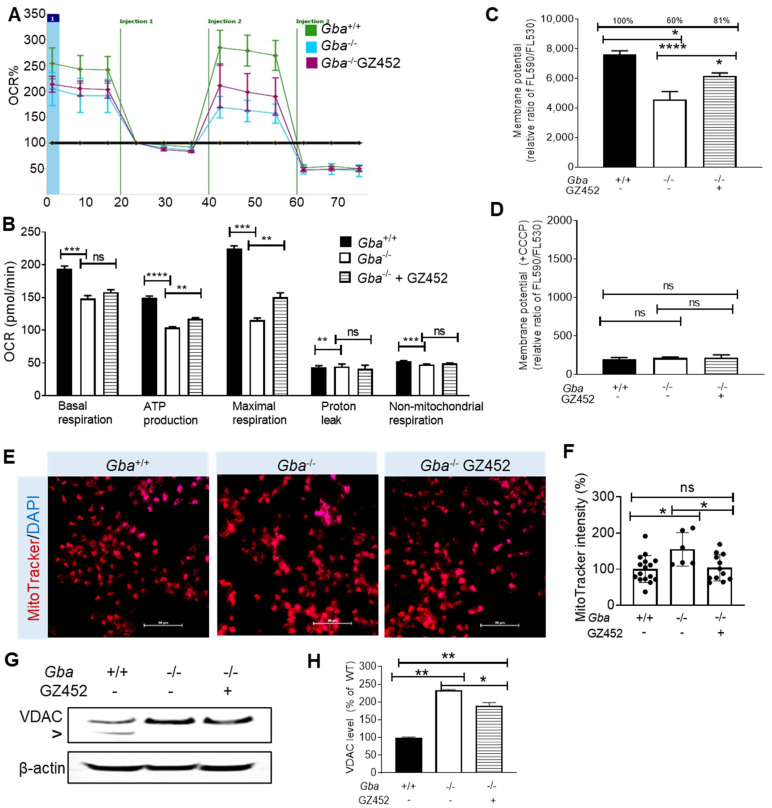
GZ452 treatment improved mitochondrial function. (**A**) Seahorse analysis of oxygen consumption rate (OCR) in *Gba^+/+^*, *Gba*^-/-^, and GZ452-treated *Gba*^-/-^ neurons. (**B**) Quantitation of OCR data showed ATP production and maximal respiration was increased in GZ452-treated *Gba*^-/-^ neurons (triplicate experiments, *n* = 4 per experiment). (**C,D**) Mitochondrial membrane potential (MMP) assay. GZ452 treatment significant improved MMP in *Gba*^-/-^ neurons (**C**). CCCP treatment disrupted MMP in neurons as a negative control (**D**) (triplicate experiments, *n* = 4 per experiment). (**E**) MitoTracker-stained mitochondria (scale bar = 50 µM). (**F**) Quantitation of MitoTracker density in cells showed reduced signals in GZ452-treated *Gba*^-/-^ neurons compared with untreated *Gba*^-/-^ neurons (*n* ≥ 6 images from triplicate experiments). (**G**) VDAC and β-actin were measured by WB. “>” points a non-specific band. (**H**) Quantitation data showed GZ452 treatment reduced VDAC protein (normalized by β-actin) level in *Gba*^-/-^ neurons (duplicate experiments). One-way ANOVA (* *p* < 0.05; ** *p* < 0.01; *** *p* < 0.001; **** *p* < 0.0001). ns, not statistically significant.

Reduced mitochondrial oxygen consumption rates (OCR) were evident in GD mice and their derived cultured cells [15,30]. OCR in the neurons was measured by Seahorse assay [30]. The results show that basal respiration, ATP production, and maximal respiration were significantly decreased in *Gba*^-/-^ compared with *Gba^+/+^* neurons (Figure 2A,B). GZ452 treatment significantly improved ATP production and maximal respiration in *Gba*^-/-^ neurons (Figure 2A,B). Mitochondrial membrane potential (MMP) is critical for maintaining the physiological function of the respiratory chain to generate ATP [32]. MMP in neurons was assayed by TMRE. Depolarized or inactive mitochondria exhibited decreased membrane potential, resulting in reduced TMRE signals, indicating MMP decline. *Gba*^-/-^ neurons showed decreased MMP to 60% of MMP in *Gba*^+/+^ neurons (Figure 2C). GZ452 treatment significantly improved MMP in *Gba*^-/-^ neurons to 81% of normal level (Figure 2C). A mitochondrial oxidative phosphorylation uncoupler CCCP dissipated MMP as a negative control in the assay (Figure 2D). 

To study if the impaired ATP production and MMP could change the mitochondrial morphology, the mitochondria were labelled by the cell-permeant MitoTracker probes that contained a mildly thiol-reactive chloromethyl moiety that passively diffused across the plasma membrane and accumulated in active mitochondria [33]. The intensity of MitoTracker signals in *Gba*^-/-^ was increased compared with *Gba*^+/+^ neurons (Figure 2E,F). Consistent with enhanced mitochondrial intensity, the mitochondrial protein VDAC (voltage-dependent anion channel), a class of porin ion channel, and Tom20 (mitochondrial import receptor subunit) located on the outer mitochondrial membrane, were increased in *Gba*^-/-^ neurons (Figure 2G and Figure S3). With GZ452 treatment, MitoTracker intensity (Figure 2E,F) and VDAC level (Figure 2G,H) were significantly reduced in *Gba*^-/-^ neurons. These results demonstrate that impaired mitochondrial function in *Gba*^-/-^ neurons can be modulated and improved by SRT treatment. 

### 3.3. SRT Protects Lysosomes and Autophagy

**Figure 3:**
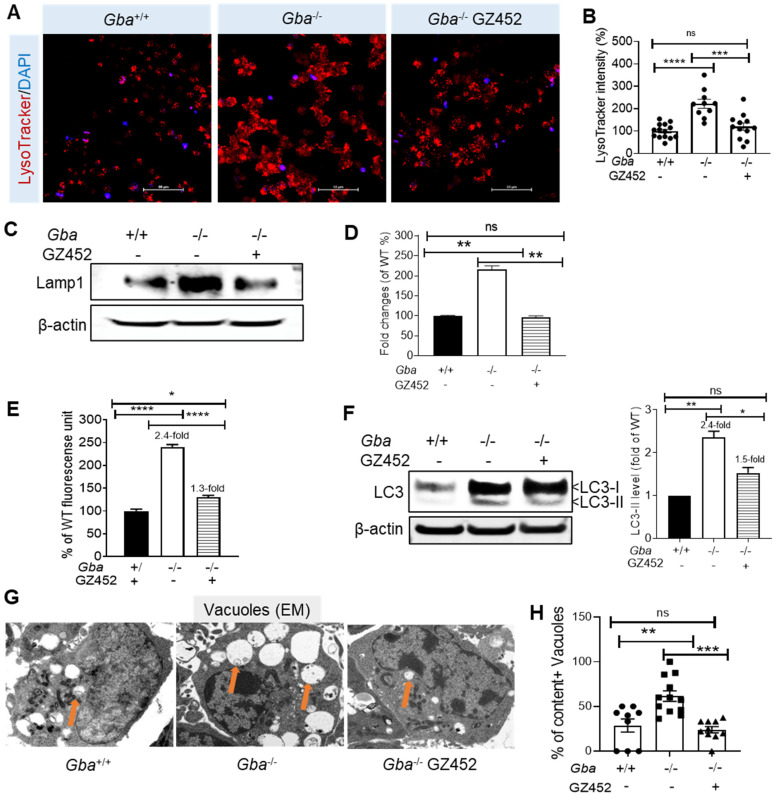
GZ452 treatment protected lysosome and autophagy in *Gba*^-/-^ neurons. (**A**) LysoTracker-stained lysosomes (scale bar = 50 µM). (**B**) Quantitation of LysoTracker density in cells (*n* ≥ 10 images). (**C**) Lamp1 and β-actin were measured by WB in neurons. (**D**) Quantitation of Lamp1 protein level after normalized with β-actin showed reduction in Lamp1 in GZ452-treated *Gba*^-/-^ neurons (duplicate experiments). (**E**) Autophagy flux was analyzed in neurons by CYTO-ID^®^ autophagy kit. Increased fluorescence unit indicates that autophagy flux was blocked in *Gba*^-/-^ neurons. GZ452 treatment improved autophagy flux from 2.4-fold to 1.3-fold above normal level (triplicate experiments, *n* = 8 per experiment). (**F**) LC3 and β-actin were measured by WB. After normalized with β-actin controls, quantitation data showed LC3-II level was increased in *Gba*^-/-^ neurons (2.4-fold) compared with *Gba^+/+^* and reduced to 1.5-fold in GZ452-treated *Gba*^-/-^ neurons (duplicate experiments). (**G**) *Gba*^-/-^ neurons contain large-size vacuoles (arrows), shown by representative EM images. Large vacuoles were not present in GZ452-treated *Gba*^-/-^ neurons. (**H**) Quantitation of vacuoles with contents in neurons (*n* ≥ 9 cells). One-way ANOVA analysis (* *p* < 0.05; ** *p* < 0.01; *** *p* < 0.001; **** *p* < 0.0001). ns, not statistically significant.

Enlarged lysosomes previously reported in *Gba*^-/-^ neurons [25] were confirmed here by enhanced intensity of LysoTracker (Figure 3A,B), a fluorescent probe highly selective for acidic lysosomes, and by increased levels of lysosomal membrane protein Lamp1 (Figure 3C,D). In GZ452-treated *Gba*^-/-^ neurons, LysoTracker intensity and Lamp1 protein levels were reduced to levels comparable with *Gba*^+/+^ neurons (Figure 3A–D). 

In lysosomal storage diseases, defective lysosomal enzyme/protein function interrupts the fusion of the lysosome with the autophagosome for proper degradation of macromolecules [34,35]. Abnormal autophagy has been demonstrated in nGD cells, animal models, and in patient brain samples [8,12,30]. The effect of GZ452 on autophagy was measured by CYTO-ID^®^ autophagy kit and autophagy marker LC3 (microtubule-associated protein 1 light chain 3). CYTO-ID^®^ autophagy kit measures autophagy flux in live cells by selectively labeling accumulated autophagic vacuoles. In *Gba*^-/-^ neurons, CYTO-ID^®^ fluorescence signal was increased 2.4-fold compared with *Gba*^+/+^ neurons, suggesting a block of autophagic flux (Figure 3E). LC3 has two forms, LC3-I and LC3II. LC3-I is distributed within the cytoplasm and nucleus. LC3-II conjugates to phosphatidylethanolamine on the autophagosomal membrane and is increased when the autophagy pathway is disrupted. *Gba*^-/-^ neurons showed increased LC3-II levels (2.4-fold) compared with *Gba^+/+^* neurons, reflecting an induction of autophagy in response to substrate accumulation-induced cellular stress (Figure 3F). Furthermore, ultrastructural analysis by EM revealed large size and increased numbers of vacuoles in *Gba*^-/-^ compared with *Gba*^+/+^ neurons (Figure 3G). Many of those vacuoles contained undigested materials, suggesting interrupted fusion of autophagosome with lysosome in *Gba*^-/-^ neurons.

The effect of SRT on normalizing autophagy was studied in GZ452-treated *Gba*^-/-^ neurons. GZ452 treatment improved autophagy flux from 2.4-fold to 1.3-fold above normal level (Figure 3E), and reduced LC3-II level from 2.4-fold to 1.5-fold (Figure 3F) in *Gba*^-/-^ neurons. The size of vacuoles was reduced (Figure 3G), and the proportion of vacuoles with undigested contents was reduced to normal levels in GZ452-treated *Gba*^-/-^ neurons (Figure 3H). These results suggest that disrupted autophagy and lysosomes are directly caused by substrate accumulation. SRT effectively rescued altered autophagy and lysosomes in *Gba*^-/-^ neurons.

### 3.4. SRT Lessened Hyperactivity of mTOR Pathway

**Figure 4:**
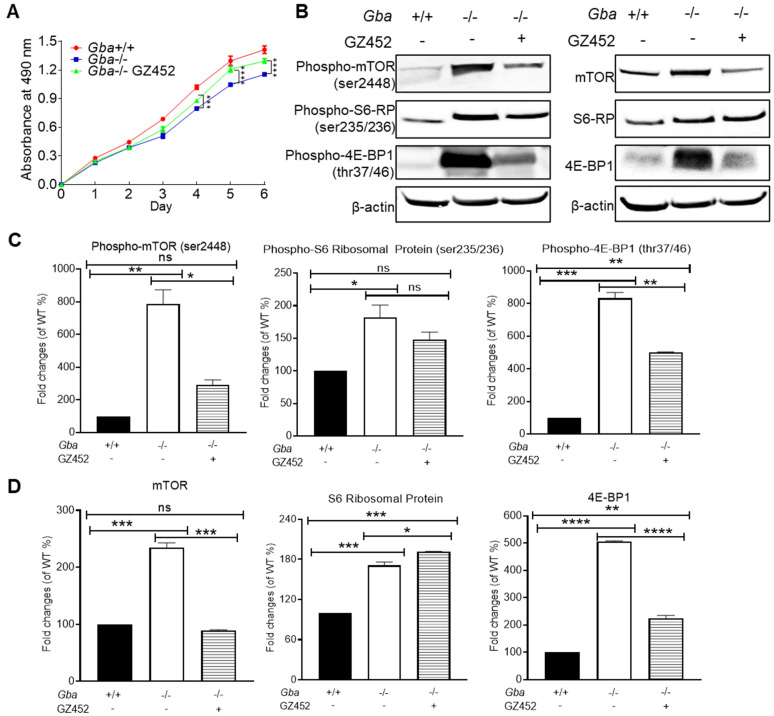
GZ452 treatment improved proliferation and reduced hyperactivity of mTOR pathway proteins in *Gba*^-/-^ neuron. (**A**) Cell proliferation was measured by MTT assay and presents as absorbance unit at 490 nm (triplicate experiments, *n* = 8 per experiments). (**B**) Protein levels of phospho-mTOR, phospho-S6 ribosomal protein (S6-RP), phospho-4E-BP1, mTOR, S6-RP, 4E-BP1, and β-actin were measured by WB. (**C**) Quantitation data (normalized by β-actin) of the protein levels of phospho-mTOR, phospho-S6-RP and phospho-4E-BP1. (**D**) Quantitation data of the protein levels of mTOR, S6-RP, and 4E-BP1. Experiments were repeated two or three times (*n* = 3 samples). One-way ANOVA analysis (* *p* < 0.05; ** *p* < 0.01; *** *p* < 0.001; **** *p* < 0.0001). ns, not statistically significant.

Defective autophagy-lysosomal function can lead to nutrition starvation, affect cell growth, and trigger hyperactivity of the mammalian target of rapamycin (mTOR) pathway in GD [18]. Indeed, *Gba*^-/-^ neurons grew slower compared with *Gba^+/+^* neurons (Figure 4A). To assess differences in cell growth between *Gba*^-/-^ and *Gba*^+/+^ neurons, cell proliferation in culture was followed for 6 days using MTT assays. Cell proliferation was reduced in *Gba*^-/-^ compared with *Gba*^+/+^ neurons. GZ452 treatment improved *Gba*^-/-^ proliferation during days 4 to 6 in culture (Figure 4A, Tables S1 and S2). 

**Figure 5:**
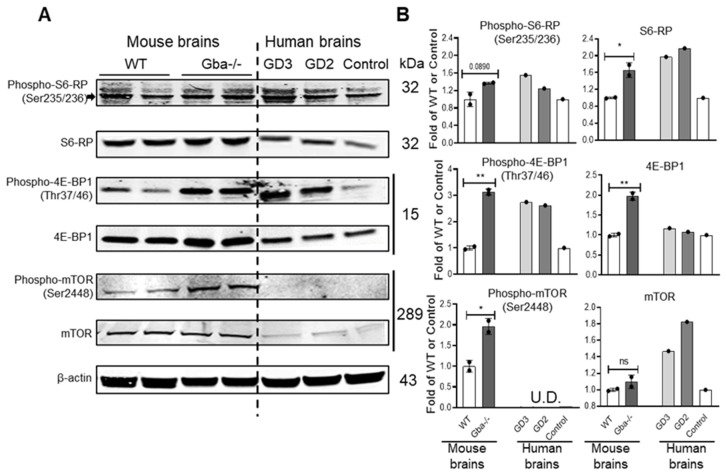
WB analysis of mTOR pathway proteins in mouse and human brains. (**A**) Protein was analyzed by WB. *Gba^-/-(NestinCre)^* mice brains (Gba^-/-^) (29 days) were compared with WT mice (35 days). Human GD2 brain (15 months) and GD3 hippocampus (11.5 years) were compared with healthy control hippocampus (12 years). (**B**) Fold change of WT level after normalization by β-actin. Phospho-S6-RP (arrow)/S6-RP, phospho-4E-BP1/4E-BP1, and phospho-mTOR were increased in *Gba^-/-(NestinCre)^* mice brains compared with WT mice. Student’s *t*-test (replicate assays, *n* = 2 mice brains). In human samples, phospho-S6-RP/S6-RP, phospho-4E-BP1/4E-BP1, and mTOR were increased in GD2 brain and GD3 hippocampus compared with healthy control hippocampus. Phospho-mTOR was undetectable (U.D.) in human brains (replicate experiments, *n* = 1 human brain). Student’s *t*-test (* *p* < 0.05; ** *p* < 0.01). ns, not statistically significant.

Activation of the mTORC1 pathway was evident in brains from nGD mice and in brains from GD2 and GD3 patients (Figure 5). In the mTORC1 pathway, mTOR regulates protein synthesis by activating S6-RP or inhibiting 4E-BP1 through phosphorylation [18,36]. WB analyses of mTOR, S6-RP, 4E-BP1, and their phosphorylated forms showed increased levels of those proteins in brains of the nGD mouse model *Gba^-/-(NestinCre)^* [26] and in autopsied brains from GD2 and GD3 patients [8] (Figure 5). The exception was phosphorylated mTOR, which was below the level of detection in human brain samples. These results provide evidence that hyperactivation of the mTORC1 pathway is a pathological phenotype in nGD brains. 

Consistent with the findings in GD brains, *Gba*^-/-^ neurons showed increased levels of mTOR, S6-RP, and 4E-BP1 and their corresponding phosphorylated proteins (Figure 4B). To determine if GZ452 treatment could diminish activated mTORC1 pathway protein, *Gba*^-/-^ neurons were treated with GZ452. This treatment reduced the level of mTOR/phospho-mTOR and 4E-BP1/phospho-4E-BP1 but did not affect S6-RP and phospho-S6-RP (Figure 4C,D). These results demonstrate that SRT, by inhibiting GC production, selectively diminishes mTOR and 4E-BP1 hyperactivities in *Gba*^-/-^ neurons, suggesting a therapeutic potential for SRT in modulating the activity of the mTORC1 pathway in GD.

TFEB is a master regulator of lysosomal gene transcription and is linked to mTORC1 function to regulate lysosomal biogenesis [37]. Cellular levels of TFEB and phosphorylated TFEB were assessed in *Gba*^-/-^ neurons. WB analyses showed increased TFEB levels in *Gba*^-/-^ compared with *Gba^+/+^* neurons (Figure S4). GZ452 treatment reduced TFEB in *Gba*^-/-^ neurons. However, the level of phosphorylated TFEB was not altered in *Gba*^-/-^ neurons or with GZ452 treatment. It was noticed that the pattern of phosphorylated TFEB in the GZ452-treated cells (single band) was different from untreated cells (multiple bands). This result suggests that SRT has an effect in modulating TFEB and phospho-TFEB levels. 

## 4. Discussion

SRT targets glucosylceramide synthase to reduce the synthesis of GC, preventing accumulation of GC and GS in GD cells harboring a dysfunctional GCase. The effect of SRT using GZ452 in CNS was demonstrated by reduction in substrate accumulation in the brain [21] as well as improved neurological function and prolonged lifespan in mouse models of nGD [22]. This study uncovers mechanisms by which SRT may protect neuronal functions in nGD. In *Gba*^-/-^ neurons, lysosomal stress due to defective GCase, and glycolipids substrate accumulations disrupted multiple cellular functions including mitochondria, autophagy, and mTOR activity. GZ452 inhibited GC production and normalized GC levels in GD neurons, leading to improved neuronal growth by enhancing mitochondrial function, restoring autophagy, and reducing mTORC1 hyperactivity (Figure S5). 

Mitochondria are involved in ATP synthesis through the tricarboxylic acid cycle and oxidative phosphorylation. Impaired mitochondrial function and energy metabolism have been implicated in nGD and other neurodegenerative diseases, including Parkinson’s disease, Huntington’s disease, amyotrophic lateral sclerosis, and Alzheimer’s disease [14,38,39,40,41]. Decreased mitochondrial oxygen consumption and ATP production are remarkable in nGD mouse brains [17,30,42]. Furthermore, reduced MMP in GD is evident in a cell model with mixed neurons and astrocytes derived from acute nGD mouse model [13,16]. These abnormalities of mitochondrial function and structure were recapitulated here in *Gba*^-/-^ neurons. MMP is an electrical gradient across the mitochondrial inner membrane. MMP drives back protons into the matrix through the F1Fo ATP synthase to generate ATP [43]. Decreased MMP in *Gba*^-/-^ neurons can affect ATP production, leading to reduced OCR. Considering the intense energy demands and limited regenerative capacity of neurons, improper functioning of mitochondria can have devastating effects on neuronal survival and can cause slow growth of the immortalized *Gba*^-/-^ neurons. 

The accumulation of pathological lipids and protein aggregates likely adds insult to MMP to compromise mitochondrial function. Previously, we reported that amyloid precursor protein aggregates co-localized with the mitochondrial membrane proteins Tom40 and Cox IV in the brain of a chronic nGD mouse model [17]. In this study, there was elevated GC associated with isolated mitochondria in *Gba*^-/-^ neurons. The excess GC in mitochondria could be transferred from lysosomes during mitochondria–lysosome contact [44]. Although we did not detect lysosomes in isolated mitochondria preparations, the mitochondria–lysosome contact is a dynamic process of intracellular communication between organelles [44]. Thus, impaired mitochondrial function (MMP, OCR) could be directly triggered by excess GC on mitochondria or indirectly by lysosome stress or overall cellular stress. Importantly, GZ452 treatment reduced GC levels in the mitochondria, improved OCR, and restored MMP to normal levels, reinforcing the detrimental impact of glycolipids accumulation on mitochondrial function, and supporting a therapeutic benefit of SRT in protecting mitochondrial function. 

Autophagic and lysosomal pathways are cellular mechanisms for recycling organelle and biological materials and for clearance of macromolecules [45]. Disturbed autophagic and lysosomal functions are major pathological bases for lysosomal storage diseases [3,46], especially in nGD [8,12]. We previously showed enhanced autophagic maker proteins (p62, LC3-II) in nGD mice and GD3 patient brains [8,12,17]. The present findings from *Gba*^-/-^ neurons support the notion of disruption of autophagic and lysosomal pathways in GD. In *Gba*^-/-^ neurons, enlarged lysosomes were associated with reduced autophagic flux and numerous massive vacuoles containing undigested materials in the cells, all indicating disrupted autophagic and lysosomal fusion. Remarkably, SRT treatment effectively reduced the enlarged lysosomes and autophagy vacuoles to restore autophagic and lysosomal pathways in GD neurons. 

In response to the stress and reduced energy production in *Gba*^-/-^ neurons, activity of the mTOR pathway was increased, and autophagic activity was deregulated, compromising normal cellular homeostasis. mTOR is a major regulator of autophagic processes, and its activity is modulated by starvation, growth factors, and cellular stressors. Here, we found that several downstream targets of mTORC1 (mTOR, 4E-BP1, and S-6RP) were hyperactivated in the brains of nGD mouse and human patients and in *Gba*^-/-^ neurons. mTOR regulates protein synthesis through the phosphorylation of 4E-BP1 and S6-RP [36]. Phosphorylation of 4E-BP1 removes its inhibition on protein synthesis, while phosphorylated S6-RP promotes translation in protein synthesis [36]. In nGD brain tissues and *Gba*^-/-^ neurons, there were increased levels of mTOR, 4E-BP1, and S6-RP proteins and their phosphorylated forms, suggesting mTOR hyperactivity in nGD. Our finding is consistent with studies in nGD iPSC-derived neurons, in which elevated GS levels were implicated in the pathological activation of mTOR [18]. In previous studies, inhibition of glucosylceramide synthase and acid ceramidase, the lysosomal enzyme that deacylates elevated GC to GS, also reduced mTOR kinase activity [19]. Overall, our results suggest that the mTOR pathway may be a potential new target for treating nGD. 

mTOR is one of the kinases that phosphorylates TFEB, the master transcription factor that regulates expression of autophagic and lysosomal genes [47]. Downregulation of TFEB protein has been shown in nGD iPSC-derived neurons [48]. In the present study, however, TFEB level was increased in *Gba*^-/-^ neurons, and the level was back to normal with GZ452 treatment, which could reflect differential TFEB activity in human and mouse GD models. Although increased level of phosphorylated TFEB (Ser142) was not detected in *Gba*^-/-^ neurons, its pattern of phosphorylation appeared to have been altered by GZ452 treatment. Thus, differences in cell type, immortalized mouse neurons vs. human iPSC-derived neurons, may account for the differences in TFEB activation we observed. The mechanism by which GZ452 affects the regulation of TFEB is an interesting question that requires further investigation.

In summary, our studies of *Gba*^-/-^ neurons indicate that the accumulation of substrates, GC, and GS is a key insult in causing disruption of cellular functions in nGD and that these excess substrates were normalized by GZ452 treatment. The complexity of disease pathology and multi-organelles dysfunction in nGD demands in-depth research that will help identify effective therapeutic approaches. The cellular mechanisms uncovered in these studies imply that SRT may help treat nGD in addition to GD1, and suggest that rescuing mitochondrial function and targeting mTORC1 pathways could be potential future therapeutics for GD. 

## Supplementary Materials

The following are available online at https://www.mdpi.com/article/10.3390/cells10092286/s1, Figure S1: GCase activity in *Gba*^*+*/*+*^ and *Gba*^-/-^ neurons, Figure S2: Optimized concentration of GZ452 for cell treatment, Figure S3: Quality of isolated mitochondria was determined by WB assay, Figure S4: TFEB and Phospho-TFEB levels in GZ452-treated *Gba*^-/-^ neurons, Figure S5: Scheme of SRT in protecting neuron functions, Table S1: A p value analysis of MTT assay, Table S2: Significant difference of MTT assay.
