# Supplementary material for: Substrate Reduction Therapy Reverses Mitochondrial, mTOR, and Autophagy Alterations in a Cell Model of Gaucher Disease
Source: Cells. 2021 Sep 2;10(9):2286. doi: 10.3390/cells10092286 (PMC8466461; doi:10.3390/cells10092286)
Supplement: Supplementary file 1 [file cells-10-02286-s001.zip › cells-1361870-supplementary.pdf]

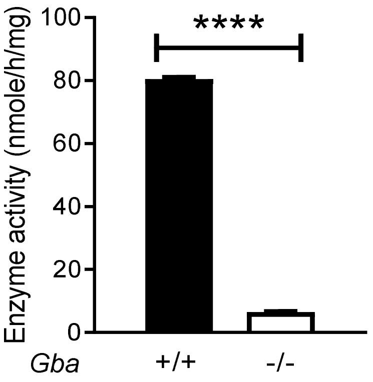

Supplementary Figure S1. GCase activity in *Gba*$^{+/+}$ and *Gba*$^{-/-}$ neurons. Experiments were repeated three times (n=5 samples). One-way ANOVA analysis (****, p<0.0001).

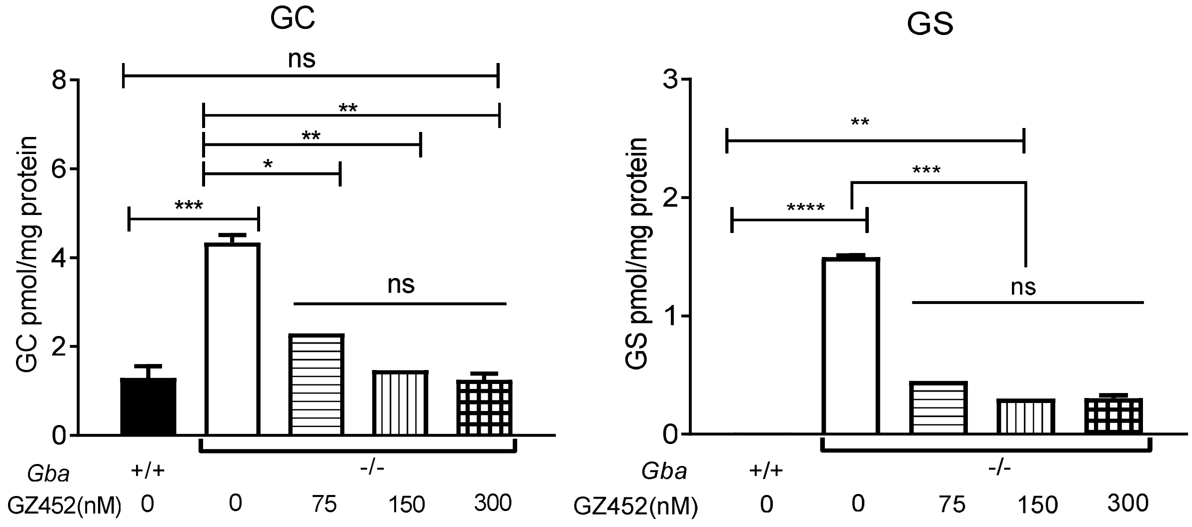

Supplementary Figure S2. Optimized concentration of GZ452 for cell treatment. *Gba*$^{-/-}$ neurons were treated with 0, 75, 150, or 300 nM GZ452 for 5 days. Substrate (GC and GS) levels were analyzed by LC/MS-MS. GC levels showed dose-dependent reduction to normal level in *Gba*$^{+/+}$ neurons. GS level was also reduced by GZ452 at three concentrations. Experiments were repeated >2 times. One-way ANOVA analysis (*, p<0.05; **, P<0.01; ***, p<0.001; ****, p<0.0001).

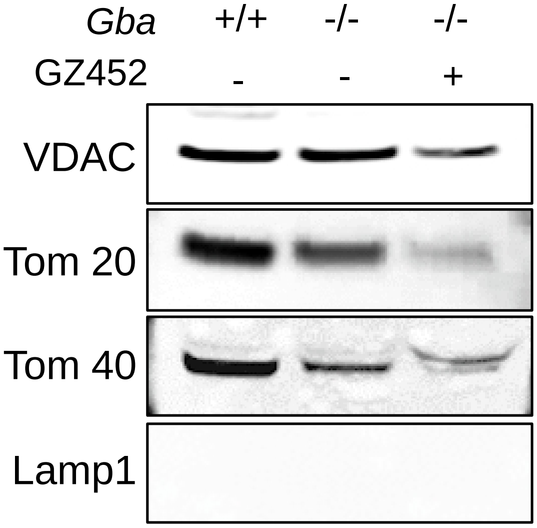

Supplementary Figure S3. Quality of isolated mitochondria was determined by WB assay. Mitochondrial markers, VDAC, Tom20 and Tom40 were detected in isolated mitochondria from *Gba*$^{+/+}$, *Gba*$^{-/-}$, and GZ452-*Gba*$^{-/-}$ neurons. The lysosome marker Lamp1 was not detected in isolated mitochondria. Experiments were repeated 2 to 3 times.

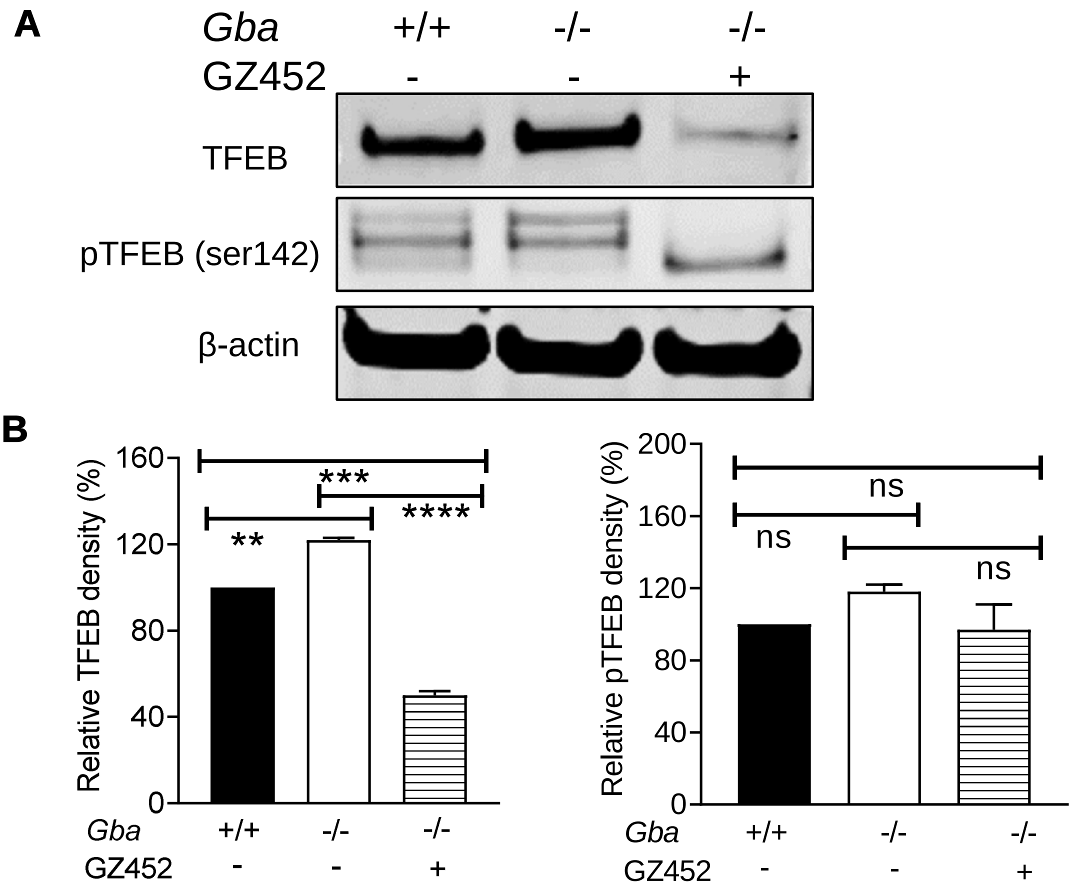

Supplementary Figure S4. TFEB and Phospho-TFEB levels in GZ452 treated *Gba*$^{-/-}$ neurons. (A) TFEB, phosphor(p)TFEB and β-actin were measured by WB. (**B**) Quantitation data was shown the protein levels of TFEB (left panel) and pTFEB (right panel). Triplicate experiments. One-way ANOVA analysis (**, P<0.01; ***, p<0.001; ****, p<0.0001).

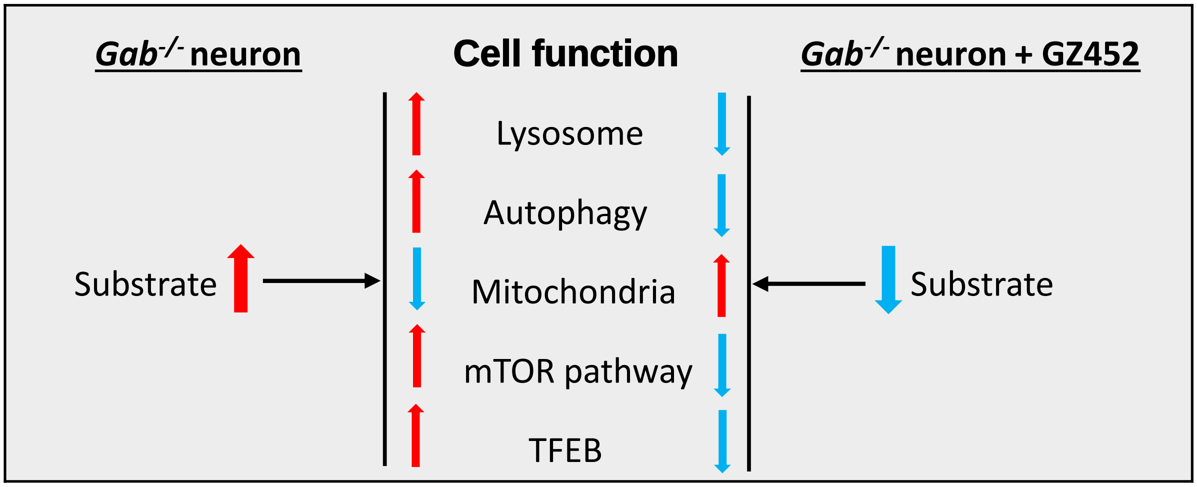

Supplementary Figure S5. Scheme of SRT in protecting neuron functions. Lack of GCase in *Gba*$^{-/-}$ neurons leads to substrate accumulation which causes lysosomal stress and affects multiple cellular functions. Impaired mitochondrial function (MMP, OCR) could be directly triggered by accumulation of substrates glucosylceramide and glucosylsphingosine or by lysosomal storage induced stress. In response to the stress and reduced energy production in mitochondria, mTOR activity and autophagy activity are increased. SRT compound (GZ452) inhibited glucosylceramide production, normalized substrate level and protected neuron growth by improving the function of mitochondria and autophagy and diminishing hyperactivity of the mTOR pathway. Red arrows indicate increase or activation. Blue arrows indicate reduction or inactivation.

Supplementary Table S1. P value analysis of MTT assay

	P value		
**Culture days**	***Gba*$^{+/+}$ VS. *Gba*$^{-/-}$**	***Gba*$^{+/+}$ VS. *Gba*$^{-/-}$ + GZ452**	***Gba*$^{-/-}$ VS. *Gba*$^{-/-}$ + GZ452**
Day 1	0.00044	0.00804	0.22572
Day 2	0.00243	0.02411	0.88220
Day 3	0.00004	0.00306	0.08627
Day 4	0.00000	0.00018	0.00418
Day 5	0.00020	0.14801	0.00019
Day 6	0.00002	0.02178	0.00015

Supplementary Table S2. Significant difference of MTT assay

	Significant difference		
	***Gba*$^{+/+}$ VS. *Gba*$^{-/-}$**	***Gba*$^{+/+}$ VS. *Gba*$^{-/-}$GZ452**	***Gba*$^{-/-}$ VS. *Gba*$^{-/-}$ GZ452**
Day 1	****	**	ns
Day 2	***	*	ns
Day 3	****	***	ns
Day 4	****	****	***
Day 5	****	ns	****
Day 6	****	*	****

Supplementary Tables S1 and S2, P value from One-way ANOVA analysis (*, p<0.05; **, P<0.01; ***, p<0.001; ****, p<0.0001).
